# Blood transfusion in haematology: A qualitative exploration of patients’ and healthcare professionals’ perceptions

**DOI:** 10.1111/bjhp.12597

**Published:** 2022-05-11

**Authors:** Brittannia Volkmer, Fabiana Lorencatto, Simon J. Stanworth, Shashivadan P. Hirani, Jill J. Francis

**Affiliations:** ^1^ Centre for Health Services Research School of Health Sciences City, University of London London UK; ^2^ Centre for Behaviour Change University College London London UK; ^3^ NHS Blood and Transplant/ Oxford University Hospitals NHS Foundation Trust John Radcliffe Hospital Oxford UK; ^4^ Oxford Clinical Research in Transfusion Medicine University of Oxford Oxford UK; ^5^ School of Health Sciences University of Melbourne Melbourne Victoria Australia; ^6^ Clinical Epidemiology Program Ottawa Hospital Research Institute Ottawa Ontario Canada

**Keywords:** Blood transfusion, cancer, haematology, outpatients, treatment decision‐making, treatment perceptions

## Abstract

**Objectives:**

Repeated blood transfusions are indicated for the management of patients with cancer or blood disorders. Patients’ perceptions about transfusions may be associated with decision‐making and coping, which has been under‐explored in the haematology context. This study therefore aimed to explore haematology transfusion patients’ and HCPs’ perceptions of blood transfusion, drawing on theory and previously identified themes of transfusion perceptions.

**Design:**

Semi‐structured interview study with 14 adult blood transfusion patients and 14 HCPs (consultants, registrars, nurses) at two UK haematology units.

**Methods:**

Patient‐ and HCP‐tailored topic guides were developed based on themes of blood transfusion perceptions identified in a systematic review: ‘Health benefits’, ‘Safety/risk’, ‘Negative emotions’, ‘Alternatives’ ‘Decision making’ and ‘Necessity’. Transcripts were analysed using deductive and thematic analysis. Patient and HCP themes were compared using triangulation methods. Conceptual models (one for patients, one for HCPs) specific to haematology portraying the association between themes were developed.

**Results:**

Findings for patients and HCPs converged with transfusion reported as beneficial for patients, who were largely involved in the decision‐making. Both groups also reported concerns about transfusion, including iron‐overload, allergic reactions and challenges to deliver transfusions in time‐pressurized services. Themes in the conceptual models included patient ‘Burden’ of receiving repeated transfusions and ‘Supportive relationships’, reflective of patients’ positive interactions with other patients and HCPs in the haematology unit.

**Conclusion:**

Despite the challenges for patients receiving repeated transfusions, convergent perceptions suggest a shared understanding of patients’ transfusion experiences. Identified challenges could inform ways to improve transfusion services and patients’ experiences.

**Statement of contribution**

***What is already known on this subject?***

- Transfusion perceptions (e.g. ‘concerns’ about risks; ‘alternatives’) may influence patient and HCP decision‐making.
- Haematology perceptions have been under investigated despite repeated transfusion use.

***What this study adds?***

- New haematology themes presented in the conceptual models inter‐link and influence ‘decision‐making’ perceptions.
- Patients report the ‘burden’ of repeated transfusions, noticeable ‘health benefits’ and the importance of ‘social connection’.
- HCPs report unique challenges with providing a high volume of transfusions, indicating the need for service reforms.

## BACKGROUND

Patients facing threats to their health (e.g., illness or symptoms) seek to understand these risks by forming illness and treatment perceptions, such as how the threat could be ‘controlled’ or managed through treatment (Leventhal et al., 1980). Considering an illness as ‘controllable’ through treatment is linked, for example with improved patient outcomes, such as quality of life (Schoormans et al., 2014; van Wilgen et al., 2008). In parallel with managing emotional responses, such as fear and anxiety, patients are likely to consider the ‘*Necessity’* of a treatment and their ‘*Concerns’* about the treatment (Horne et al., 2013). Patients may hold concerns, for example if they associate a potential treatment with a certain level of risk. Blood transfusion is a very common procedure in hospitals and represents an example of an intervention where patients face potentially significant risks from the transfusion, such as infection transmission or adverse reactions to the blood products (Serious Hazards of Transfusion (SHOT), 2021).

In the United Kingdom (UK), around two million blood components were issued from the UK Blood Services in 2020 with the main indications for treatment being severe bleeding or to replace blood loss due to conditions affecting blood cell production (e.g., bone marrow failure, anaemia) (Narayan et al., 2021). Despite transfusion being used for patients worldwide (around 3.5 million patients across the European Union (25 million units of blood)) (European Commission, 2018), there has been little exploration of patients’ and health care professionals’ (HCPs’) perceptions of blood transfusion. A recently published systematic review (Abdul‐Aziz et al., 2018) synthesized 32 globally derived studies and identified that patients and HCPs viewed transfusion as having low‐to‐moderate risk. Some perceptions of transfusion‐associated risk (e.g., infections or reactions), however, were associated with the consideration and preference for transfusion alternatives, such as ‘monitoring’ instead of transfusion or transfusing with the patient's own pre‐operative stored blood (Abdul‐Aziz et al., 2018). Some patients viewed transfusion as having benefits (Davis et al., 2012; Murphy et al., 1997), but others reported that benefits were difficult to discern due to the impact of their illness (Fitzgerald et al., 1999; Orme et al., 2013). It was also reported that HCPs led the decision‐making process (Adams & Tolich, 2011).

From the systematic review's inductive synthesis of the reported perceptions, five themes related to blood transfusion treatment perceptions were identified: ‘Safety/risk’, ‘Alternatives’, ‘Health benefits’, ‘Negative emotions’ and ‘Decision making’ (Abdul‐Aziz et al., 2018). These themes are conceptually similar to questionnaire items commonly used to assess patients’ treatment perceptions; The Beliefs about Medicines Questionnaire (BMQ) (Horne et al., 1999b, 2004) and similar to constructs in the extended Self‐Regulation model of treatment perceptions, in that they consist of both cognitive and emotional representations (Horne, 2003). Although transfusions are a distinctive form of treatment (e.g. non‐chemically derived substance with fewer adherence challenges), perceptions about transfusion are likely to be aligned with existing classifications of treatment perceptions more generally.

Whilst we have a good understanding of patients’ and HCPs’ perceptions of blood transfusion, these are largely limited to surgical contexts, where transfusion is one‐off (Abdul‐Aziz et al., 2018). Around two‐thirds of all red blood cell transfusions are used medically, including for the treatment of patients with anaemia related to chronic blood disorders such as thalassaemia or cancer (e.g., Leukaemia, Lymphoma and multiple myeloma) (Euractiv, 2020; NHS Choices, 2022). Haematology patients regularly and frequently receive blood transfusions for example four hours receiving one unit (bag) of blood per week (NHS Choices, 2022). This is likely to mean that their perceptions are different to one‐off transfusions in surgical contexts. Repeated transfusions are potentially more burdensome and anxiety provoking due to the frequency that transfusions are required (Trachtenberg et al., 2012). Yet, some haematology (Myelodysplastic) patients in Sweden have been reported to perceive positive effects from transfusion, such as improved strength and mood post‐transfusion (Ryblom et al., 2015), indicating that a range of perceptions may exist in this clinical context.

### Study aims

This study aimed to explore haematology transfusion patients’ and HCPs’ perceptions of blood transfusion, drawing on theory and previously identified themes of transfusion perceptions. The study addressed the following research questions:

1. What are haematology patients’ and HCPs’ perceptions of blood transfusion?
2. To what extent do patients’ and HCPs’ perceptions align with previously published themes of blood transfusion perceptions?
3. How comparable are patients’ and HCPs’ perceptions?

## METHODS

### Study design

Semi‐structured interview study applying directed content analysis (Hsieh & Shannon, 2005), inductive thematic analysis (Braun & Clarke, 2006) and triangulation methodology (Farmer et al., 2006; Hopf et al., 2016) to generate and compare patient and HCP themes.

Few studies have used qualitative methods to explore transfusion perceptions (Abdul‐Aziz et al., 2018) meaning that we lack in‐depth understanding of participants’ perceptions and experiences.

### Setting and participants

Haematology patients and HCPs from NHS haematology day units were jointly included in the study to understand both groups’ perceptions in parallel, with few studies having previously explored both groups’ perceptions concurrently (Abdul‐Aziz et al., 2018). HCPs are a key part of a transfusion, often responsible for transfusion decision‐making on behalf of the patient and their perceptions may not align with those of patients (Morita et al., 1999). This may prevent treatment goals from being readily established and perceptions being discussed openly, leading to misunderstandings (Barry et al., 2000; Mead & Bower, 2000).

The specific patient and HCP inclusion criteria for this study were:

‐ Adult patients (aged 18+) with a non‐acute haematological disorder attending UK NHS haematology day units for a blood transfusion were eligible for inclusion. Patients were excluded if they had limited English or cognitive impairment.
‐ HCPs, including consultants, physicians and haematology nurses, working in the same haematology unit as the recruited patients, who discussed or delivered transfusions to their patients, were eligible for inclusion.

Ethical approval was granted from South Central – Hampshire B Research Ethics Committee (15/SC/0757).

### Recruitment

An on‐site member of staff facilitated recruitment and informed each eligible patient attending for a transfusion about the study providing a Participant Information Sheet (PIS). The study was also verbally promoted to HCPs in the haematology units by the site Investigator and a HCP‐specific PIS was provided. A minimum of 13 patients and 13 HCPs was the target sample size following data saturation sample size guidance (Francis et al., 2010). Interviews after the tenth interview per group (e.g., 11th, 12th…) were assessed during data coding to determine whether additional themes could be created or whether the themes were completed and sufficiently populated.

### Topic guide

Patient‐ and HCP‐specific topic guide questions were structured around the themes of blood transfusion perceptions identified in the review: ‘Safety/risk’, ‘Alternatives’, ‘Health benefits’, ‘Negative emotions’ and ‘Decision making’ (Abdul‐Aziz et al., 2018), in addition to the construct of ‘*Necessity’* (Appendix 1). This construct was added as it is central to the understanding of treatment perceptions, defined as ‘the perceived role of [the treatment] in protecting against deterioration of the present and future health status of the patient’ (Horne et al., 1999b). The topic guides were developed in collaboration between psychologists, a patient representative and Consultant Haematologist, and piloted prior to data collection.

### Procedure

Participants were interviewed individually, face‐to‐face, in the haematology unit or by telephone if preferred. For face‐to‐face interviews, patients were offered to be interviewed before, during or after their transfusions in the haematology unit. A demographic questionnaire was used to collect participant demographic data. All participants provided informed consent before interview. Interviews lasted between 30 and 45 min and were audio‐recorded, transcribed verbatim and fully anonymised. Informed consent was not collected from participants to share anonymised transcripts, therefore transcripts were unable to be uploaded to open access repositories.

### Analysis

Transcripts were first analysed using a deductive directed content analysis approach (Hsieh & Shannon, 2005), with participant responses coded into the blood transfusion themes that they were judged to best represent (Abdul‐Aziz et al., 2018). For example, a quote ‘*patients benefit from transfusions to restore their energy’* was coded to the theme ‘Health benefits’ or ‘*I do worry less about how risky the blood is nowadays’*, coded to ‘Negative emotions’ and ‘Safety/risk’. Perceptions that could not be coded into existing themes were coded into a temporary ‘other’ category. Two researchers independently coded one patient and one HCP transcript (7% of total), and inter‐rater reliability assessed using percentage agreement with >75% considered an acceptable level of agreement (Stemler, 2004).

Inductive thematic analysis was subsequently performed to generate subthemes for data deductively coded to each transfusion theme. Content coded to the ‘other’ category were also developed into themes, identified as patterned responses (Braun & Clarke, 2006) to describe the blood transfusion perceptions reported by patients and HCPs. This involved generating initial codes for interesting features of the data and collating codes into potential themes, which were then reviewed and named (Braun & Clarke, 2006). Data saturation was assessed by BV during coding to determine that the final three interviews from each group produced no new themes (Francis et al., 2010).

Themes between patients and HCPs were compared using a triangulation approach (Farmer et al., 2006; Hopf et al., 2016). For this, a convergence matrix was generated and subthemes were tabulated as either: ‘*agreement’*, a comparable subtheme present for both groups; ‘*partial agreement*’, partially comparable subthemes; ‘*disagreement’*, a contradictory finding between subthemes or ‘*silent’*, subtheme recognised by only one group.

Final themes were organised into patient‐ and HCP‐specific conceptual models of blood transfusion perceptions, specific to the haematology context.

## RESULTS

### Participant characteristics

Fourteen patients (eight from site 1, six from site 2) and 14 HCPs (seven from site 1, seven from site 2) were included in the study out of 59 patients and 43 HCPs who were informed about the study from both sites and invited to participate. Participant demographics are presented in Tables 1 and 2. Red blood cell transfusions were provided to 93% of patients and 50% had previously received platelets. Thirteen patients were interviewed during transfusions and one interview started pre‐transfusion.

**TABLE 1:** Patient demographics

Patients included (*N* = 14)	
Gender	
Male	6 (43%)
Female	8 (57%)
Ethnicity	
White English/Welsh/Scottish/Northern Irish/British	10 (71%)
Any other White background	1 (7%)
Asian/Asian British	1 (7%)
African	1 (7%)
Any other ethnic group	1 (7%)
Highest level of education completed	
No formal education	3 (21%)
A‐Levels/college certificate	2 (14%)
Graduate/professional	2 (14%)
GCSE / O‐Levels	4 (29%)
University level	3 (21%)
Haematological conditions	
Myelodysplasia	3 (21%)
Myeloma	1 (7%)
Myelofibrosis	1 (7%)
Lymphoma (CLL) and a second haematological condition	1 (7%)
Acquired haemolytic anaemia	1 (7%)
Inherited anaemia, including Thalassemia	2 (14%)
Aplastic Anaemia	3 (21%)
Other Anaemia	2 (14%)
Age	
<45	1 (7%)
45–65	4 (29%)
>65	9 (64%)
Religion	
Christian	11 (79%)
No religion	1 (7%)
Prefer not to provide	2 (14%)

**TABLE 2:** Healthcare professional demographics

Healthcare professional participants	*N* = 14
Gender	
Male	5 (36%)
Female	9 (64%)
Clinical role	
Consultant Haematologist	3 (21%)
Specialty doctor (Haematology)	1 (7%)
Specialist Registrar	2 (14%)
Specialist House Officer	1 (7%)
Senior Charge Nurse	1 (7%)
Haematology Specialist Nurse	2 (14%)
Nurse (other)	2 (14%)
Transfusion Practitioner	1 (7%)
Clinical Psychologist	1 (7%)
Interaction with patients in the haematology unit	
Daily (discussion and blood product administration)	2 (14%)
Weekly (discussion and blood product administration)	3 (21%)
Daily discussion	4 (29%)
Weekly discussion	3 (21%)
Monthly discussion	2 (14%)

### Reliability of perceptions and data saturation

Inter‐rater agreement for coding decisions was 67% for patient transcripts and 60% for HCP transcripts. Full consensus was reached on all disagreements.

Data saturation was reached at the 11th patient and 11th HCP interview with no new themes identified beyond this point. Yet due to the 12th and 13th participants in both groups continuing to provide rich data, populating the themes further, and additional 14th interview for both groups was conducted and included in the analysis being assessed for saturation (Appendix 2).

### Patients’ perceptions of blood transfusion

Perceptions were coded deductively into all six blood transfusion themes: ‘Health benefits’, ‘Necessity’,’Negative emotions’, ‘Alternatives’ and the re‐titled constructs of ‘Awareness of risk/Safety’ and ‘Involvement in decision making’. Three new themes were developed from data initially coded to ‘other’: ‘Social connection’, ‘Burden’ and ‘Distinguishing between blood products’ (Tables 3 and 4 provides sample quotations with a full list of themes provided in Appendix 3).

**TABLE 3:** Example quotations for haematology patient themes

Haematology patient themes – example quotes
*Awareness of risk/safety:* ‘there's always situations that I was here hours waiting for blood and I ended up receiving it, but the following day I was in bed all day’ (Patient 12, Aplastic Anaemia, Site 2)
*Health benefits:* ‘well normally it/ well, it depends, erm, how low the haemoglobin is, the lower it is then the benefit is quicker because you're being topped up but’ (Patient 14, Inherited Anaemia, inc Thalassemia, Site 2)
*Necessity:* ‘oh yes, it just seems to me as being essential, erm, as much as say needing oxygen in the air is an essential, without the transfusions I wouldn't be here’ (Patient 4, Aplastic Anaemia, Site 1)
*Negative emotions:* ‘so, it doesn't look, it looks ok for you for a minute, and then you start to think about something else but that one you have to be on the positive side all the time, maybe not keep saying ‘that's not good'” (Patient 9, Acquired Haemolytic Anaemia, Site 2)
*Alternatives: ‘*I was offered the main treatment for this condition, is the bone marrow transplant, but I, I'm not really keen to do that, cause it's, I've, erm, you know, the side effects of treatment …’ (Patient 12, Aplastic Anaemia, Site 2)
*Involvement in decision‐making*: ‘yeah I had choice, I had a choice, yes I will go along with it, or no I won't bother. She gave me that choice as well, ∙ but I was led by her professional advice’ (Patient 1, Myeloma, Site 1)
*Social connection ‘*I’ve met, half a dozen people over the course of the time and er, but they all have/ they either have/ they've all got some sort of cancer treatment or some sort of deficiency, but it's not the same as my own, but even if it were, I’m not sure that would take me very far, er, swapping notes with someone else whose got erm … wouldn't really give me any great comfort or distress’ (Patient 4, Aplastic Anaemia, Site 1).
*Burden:* ‘I carry on completely normal, normal life with the, you know, the odd transfusion every now and again, yeah’ (Patient 8, other Anaemia, Site 1)
*Distinguishing between blood products: ‘*it's irradiated blood like I told you … it's just blood really’ (Patient 11, Lymphoma (CLL) and a second haematological condition, Site 1).

**TABLE 4:** Example quotations for haematology HCP themes

Haematology HCP themes – example quotes
*Awareness of risk/safety:* ‘…it's all quite safe, erm, in terms of, em, getting the right blood product for the patient, cause it's different steps in safeguarding steps to do that’ (HCP 10, Senior House Office, Site 2)
*Health benefits:* ‘Yeah, so largely it erm, taking away the tiredness and lethargy, which is, er, a symptom of patients that are anaemic, er, and for some of them improving their symptoms of shortness of breath on exertion’ (HCP 7, Specialist Registrar, Site 1)
*Necessity: ‘*as I've said, it's the only way, with a lot of these people, it's the only thing that's keeping them alive, or it's the thing that's allowing them to have treatment, that's hopefully going to keep them alive’ (HCP 8, Nurse, Site 2)
*Negative emotions:* ‘it is worrying, I mean people, if they've got cardiac problems, they can get chest pain, just really really unwell’ (HCP 2, Nurse, Site 1)
*Involvement in decision‐making*: ‘… other patients want to take control of it and not be told what to do, so you have to be, erm, I think you have to be flexible about that’ (HCP 7, Specialist Registrar, Site 1)
*Alternatives:* ‘most of these patients that are on such regular transfusion programmes are on EPO (erythropoietin) are on iron, are on all the other kind of alternatives to blood that they can be on and despite that are still requiring a blood transfusion’ (HCP 10, Senior House Office, Site 2)
*Burden:* ‘because these patients if they don't have their transfusions, they aren't able to get up and be, have that level of activity that they would have without that transfusion, so despite having to come in and have a cross‐match one day, blood the following day and then only really have one day out of hospital a week, they still manage to maintain some quality of life’ (HCP 14, Specialist Registrar, Site 2).
*Organizational factors:* In relation to the complexity of managing the slots: ‘we'll sit down and we'll look and “well if I have to cancel this person this week, if they needed a transfusion in three weeks” time, are we going to be able to have a slot?’ you know, that sort of thought behind it, so it's quite complicated … we try and stretch it because of the lack of availability of slots, we try and stretch it to the absolute maximum, so we/ and we try not to prescribe if we don't have to prescribe’ (HCP 3, Specialty Doctor, Site 1).
*Stability and variability of transfusion perceptions: ‘*before blood transfusions were a one‐off sort of thing … since being here, it's actual haematology conditions that require regular blood transfusions, so I see a different side now, and I see, erm how reliant people are on them’ (HCP 5, Consultant Haematologist, Site 1)
*Supportive relationships: ‘*Erm, some of them might get some benefit out of that [lengthy appointments] cause it gives them an opportunity to have a chat with patients who are going through a similar experience and see that they're not alone’ (HCP 6, Consultant Haematologist, Site 1).

#### Awareness of risk/safety

Patients recounted negative consequences that they experienced during and after transfusion, mainly from painful cannulisation and beliefs about adverse effects; high iron levels risking organ damage. Some patients reported that reactions were only likely at the start of the transfusion or had no negative consequences to report.

#### Health benefits

Transfusions aided patients to keep going with their daily lives, reducing tiredness, improving blood levels and well‐being. Many patients were persuaded of the benefits by HCPs or significant others and thought that other patients must also perceive their transfusions as beneficial. Two patients commented that the benefit was variable and can take time to be felt or that having transfusions left them feeling drained.

#### Necessity

Patients reported that their transfusions were a supportive treatment for immediate health needs (e.g., to support chemotherapy) or for long‐term condition management. Some patients recognised that they needed transfusions through their symptoms (e.g., lethargy; *n *= 8). Other patients relied on HCPs and clinical indicators to determine the transfusion necessity, being often problematic for patients to establish a transfusion routine.

#### Negative emotions

Some patients reported worries, fears and frustrations with receiving transfusions or found them hard to initially deal with. Some patients disliked being cannulated or the time length of the transfusion. Despite this, a greater number of patients discussed having no concerns with transfusion or that any negative emotions were managed by communicating with HCPs or keeping a hopeful and positive outlook. Some patients liked attending for transfusion, expressing gratitude that blood was available.

#### Alternatives

Patients discussed alternative treatments (e.g., tablets: *unspecified*) trialled to reduce their transfusion requirement, or previous unsuccessful or non‐feasible treatments (e.g., bone marrow transplant). Some patients reported preferring, yet lacking, an alternative to transfusion. Other patients commented that their body may naturally, with time, restore depleted blood cells.

#### Involvement in decision‐making

Patients reported either being involved in the initial transfusion decision‐making, or that they deferred decisions to HCPs, or that HCPs solely made the decisions. Many patients accepted HCPs’ decisions, finding transfusion discussions positive or had limited or no choice but to accept the transfusion. For some patients, transfusions were almost an ‘automatically’ prescribed treatment.

#### Social connection

Six patients reported interactions with other patients in the unit and were positively involved in their transfusions (e.g., through asking HCPs general questions or to discuss their transfusion regimens). Some patients reflected on wider family and General Practitioner (GP) support or talked of a curiosity or connection to the blood donors. Yet, other patients felt that they lacked or had limited interaction with other patients, especially patients with different haematological conditions or to respect their privacy, feeling that greater involvement was complex.

#### Burden

Half of the patients interviewed reported that transfusions were a part of their routine life, that attendance was not a great burden and that the experience was consistent over time and easy (switching between red cell and platelet transfusions). Around an equal number of patients (*n *= 8) reported, however, that transfusions were inconvenient, involving lengthy appointments, frequent hospital trips and restriction to activities such as travelling.

#### Distinguishing between blood products

Four patients shared perceptions about specific blood products, mostly platelets. These patients perceived platelets fairly positively (e.g., quicker to infuse) and had less curiosity over their donor origin. However, there were some knowledge gaps about platelets; what platelets did and where they were harvested from, and one patient specifically reported that she was receiving irradiated blood, with no greater elaboration on the distinction of this.

### Conceptual model of patients’ haematology blood transfusion perceptions

**FIGURE 1:**
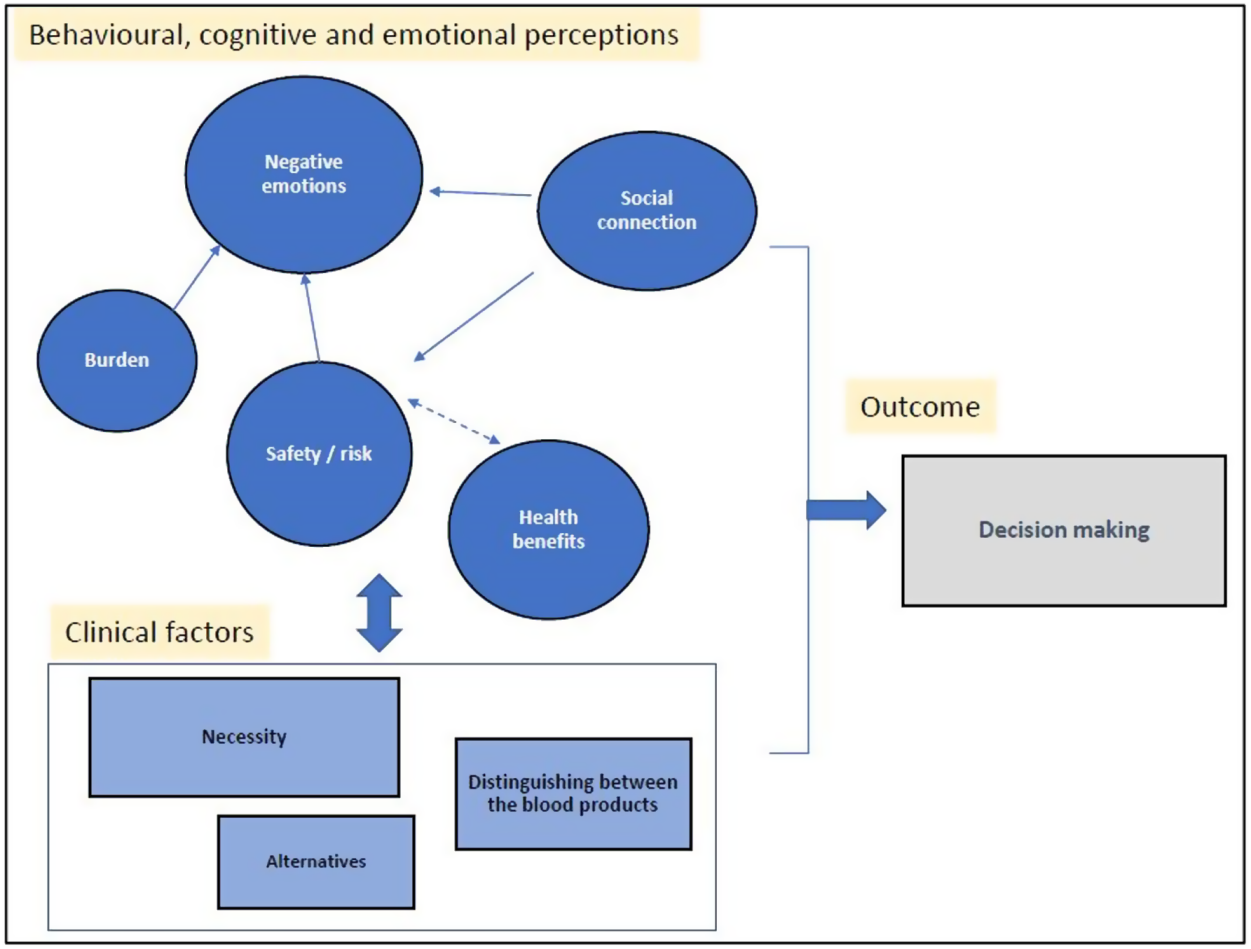
Conceptual model of patients’ haematology blood transfusion perceptions. *Note*: Single headed arrows indicate a direct one‐way relationship, double headed dashed arrows indicate a bi‐directional relationship between themes. Influences on ‘Decision making’ are shown using the central arrow

In the patient’s model (Figure 1), ‘Burden’ and ‘Safety/risk’ are proposed to be linked with ‘Negative emotions’ due to some patients reporting their transfusions to be time‐consuming or involving risk. ‘Health benefits’ and ‘Social connection’ are associated with perceptions in the ‘Safety/risk’ theme, due to patients often deliberating risk vs. benefit and efforts made to have positive social interactions (e.g., with HCPs) reportedly eased some patients concerns or information needs.

The ‘Clinical factors’ box displays three themes that impact on the behavioural, cognitive and emotional perceptions, such as the availability of alternatives, differences in perceptions depending potentially on the blood product being transfused and the transfusion ‘Necessity’ determined by HCPs or clinical indicators. In total, all themes are influences on ‘Decision‐making’, such as transfusion agreement (patient consent) or HCP transfusion prescription.

### Health care professionals’ perceptions of blood transfusion

HCPs’ perceptions were coded deductively into all six existing blood transfusion themes and four themes were additionally identified: ‘Burden’, ‘Organisational factors’, ‘Stability/Variability of transfusion perceptions’ and ‘Supportive relationships’. A full list of themes is provided in Appendix 3.

#### Awareness of risk/safety

Half of the HCPs interviewed reported iron overload as the key risk for this patient group and HCPs widely discussed risk management through safe transfusion and blood testing practices. Patients were informed of risks such as iron overload, antibody production, infections or allergic reactions, as well as risks associated with non‐transfusion verbally by HCPs. A few HCPs identified other medical and psychological risks with transfusions, such as bone marrow damage, venesections, nausea or patient dependency on transfusions.

#### Health benefits

HCPs reported that transfusions improved patients’ symptoms and helped them to maintain their quality of life and daily activities. HCPs discussed transfusion benefit with patients to continue their transfusion yet were aware that the experienced benefit for patients will end at some point. A number of HCPs felt that their colleagues shared their view of transfusion being beneficial, yet some risk‐benefit considerations in particular patient cases were questionable.

#### Necessity

HCPs reported that transfusions aided patient survival and protected their health, (e.g., when receiving chemotherapy or suffering from anaemia). HCPs reported prescribing transfusions based on clinical factors, such as haemoglobin levels and patient functioning. HCPs reported that some patients who depend on their transfusions experienced anguish when transfusions are deferred or delayed.

#### Negative emotions

HCPs expressed concern about the burden and physical impact of transfusion for their patients, with safety procedures reducing some of this concern and HCPs experienced capacity pressures. Despite this, HCPs offered patients reassurance to help reduce their anxieties, feeling that patients’ fears may inhibit their involvement in transfusion discussions. HCPs found that some patients expressed positive emotions when transfusions were not required or viewed transfusions as a ‘lifeline’.

#### Involvement in decision‐making

The majority of HCPs reported advocating for and involving patients in transfusion decision‐making despite some decisions being made without patient involvement. Many HCPs made transfusion decisions with other HCPs, laboratories or used guidelines and transfusions were often part of a broader treatment plan. A slight tendency was reported towards providing transfusion if symptoms indicated so, but patients did often question decisions or resisted transfusions.

#### Alternatives

HCPs supported greater consideration into the use of alternatives and consulted patients who may request alternatives. Many alternatives, however, were either being tried to some benefit (Erythropoietin injections) or alternatives generally were not an option for this patient group. Some HCPs understood that patients may prefer alternatives, but opted for transfusion due to symptoms, clinical indicators or the evidence base.

#### Burden

The majority of HCPs anticipated that frequent and lengthy appointments must be burdensome for patients, and seven HCPs commented that transfusions have become a part of patients’ routine lives. One HCP commented that patients could maintain some quality of life despite frequent hospital visits.

#### Organisational factors

Many HCPs from both sites acknowledged constraints to fully discuss patients’ transfusion perceptions, such as busy haematology units. HCPs clarified the complexity of managing limited transfusion slots and high and costly blood use, feeling that solutions were needed to improve processes and ease capacity strain. Patient‐ or HCP‐education was signalled as potentially required to enhance communication.

#### Stability and variability of transfusion perceptions

Many HCPs reported that their views about transfusion were stable over time and similar to their colleagues. Their views did, however, develop with haematology exposure with HCPs recognising their increased tolerance of lower haemoglobin levels and broadening knowledge about the varied and long‐term use of transfusions.

#### Supportive relationships

HCPs discussed how they remained approachable to patients and established bonds by listening to patients and providing information like blood counts, enhancing patients’ feelings of control. HCPs aimed to increase patients’ level of comfort during the transfusions, or patient support was provided from other patients or patients’ acquaintances whom they attended the transfusion with.

### Conceptual model of health care professionals’ haematology blood transfusion perceptions

**FIGURE 2:**
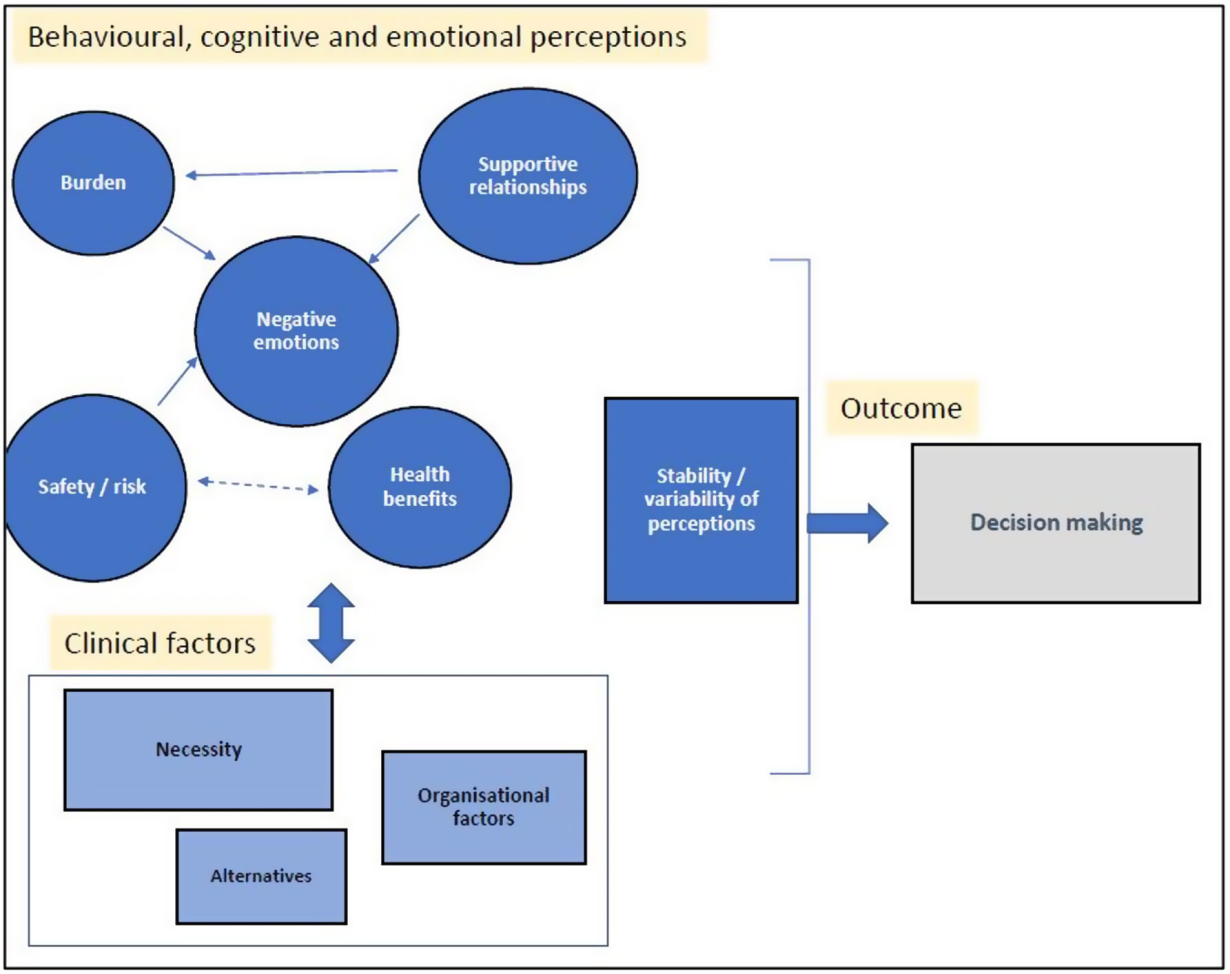
Conceptual model of HCPs’ haematology blood transfusion perceptions. *Note*: Single headed arrows indicate a one‐way relationship and double headed arrows indicate a bi‐directional relationship between themes. Influences on ‘Decision making’ are shown using the central arrow

In the HCP haematology conceptual model (Figure 2), three themes are proposed to link directly with ‘Negative emotions’: transfusion ‘Burden’, ‘Safety/risk’ concerns and ‘Supportive relationships’, which could help ease patients’ concerns or worries. ‘Supportive relationships’ such as positive contact with HCPs and other supportive contacts, whilst patients receive their transfusions may also ease perceptions of transfusion ‘Burden’. ‘Safety/risk’ vs. ‘Health benefits’ decisions are associated, being routinely considered and discussed with patients and the model also displays ‘Clinical factors’ which influence ‘Decision making’. This is transfusion ‘Necessity’, the suitability of ‘Alternatives’ and ‘Organisational’ appointment availability etc. Prior to decision‐making, the ‘Stability or variability of HCPs’ transfusion perceptions may influence tolerance of (lower) haemoglobin levels, practice consistency, transfusion acceptability and team decision‐making agreement.

### Triangulation of themes from patients’ and health care professionals’ perceptions

There was moderate convergence of the 95 patient and HCP subthemes (42%). Full agreement was evident on 24 occasions (35%), partial agreement on seven occasions (8%), disagreement on four occasions (5%) and silence on 39 occasions (52%). Appendix 4 lists all themes with their agreement ratings.

Agreement was greatest for the ‘Health benefits’, ‘Alternatives’ and ‘Involvement in decision‐making’ themes, for example HCPs reported similar benefits of blood transfusion as patients. Partial ratings occurred through HCPs reporting patient‐involvement in their transfusion decisions, yet some patients commented that although involvement was generally positive, transfusions were often not discussed. Disagreement in themes occurred when patients expressed having life restrictions (‘Burden’) in respect to travel, whereas some HCPs reported that transfusions can become a part of a patient's routine life.

Silence (i.e., theme was evident for one sample but not the other) occurred for themes, in which patients or HCPs expressed their particular patient/HCP role experiences (e.g., no negative consequences from transfusion (patients) or that patients are reviewed and consulted with when transfusions may need to end (HCPs)).

## DISCUSSION

This qualitative interview study found that haematology patients and HCPs considered transfusions beneficial for managing patients’ haematological disorders, with HCPs and patients working together to discuss transfusion decisions, risks and alternatives. However, both patients and HCPs experienced negative emotions, linked to the potential harms and ‘Burden’ of transfusion for patients; HCPs also reported concerns regarding constraints with transfusion services. Strategies to manage negative emotions were reported as remaining positive and hopeful (patients) or offering reassurance to patients or trusting clinical safety measures (HCPs).

There was moderate convergence between patients’ and HCPs’ perceptions, reflective of the groups having different roles (patients vs. providers). For example, HCPs’ perceptions often exemplified their compassionate patient care: (‘Supportive relationships’), within a busy and pressurised environment: (‘Organisational factors’) whilst patients were able to reflect on their interaction with other patients and HCPs (‘Social connection’).

The findings from this study extend previously identified themes related to blood transfusion treatment perceptions (Abdul‐Aziz et al., 2018), with ‘Decision making’, for example, being a key theme. In contrast to other findings, many patients in this study did not find their transfusions to be overly anxiety provoking or hard to come to terms with (Randall & Wearn, 2005). Instead patients remained hopeful, keeping a positive outlook, similar to other haematology patients who reported acceptance of their conditions, changing life's priorities, and increasing engagement with HCPs and significant others as a way to cope with their conditions (Bulsara et al., 2004) (Prip et al., 2017). Patients may have used appraisal‐focussed coping to accept the reality of their situation, redefining it as acceptable (Moos & Schaefer, 1984), similar to dialysis patients who perceive dialysis as a life‐sustaining gift (Reid et al., 2016).

Some patients in this study reported limited interaction whilst in the transfusion units with both other transfusion patients and HCPs. With HCPs there was often a lack of involvement in the transfusion decision‐making, as also cited in the broader cancer literature (Bruera et al., 2001). However, other patients enjoyed attending for their transfusions, with some not requiring extensive interaction with other patients. Such patients are likely to have developed ways that they cope with their transfusion, such as occupying themselves during their transfusions and engaging with patients and HCPs as and when available. As described in the Common Sense Self‐regulation Model, the positive outcomes of such strategies inform the development of new perceptions (Leventhal et al., 1980, 1984), which is evidenced with patients discussing positive emotions, such as gratitude.

This research adds perceptions of blood transfusion as a life‐sustaining intervention to the treatment perceptions evidence base, similar to perceptions of other repeated treatments, such as asthma medications (Østrem & Horne, 2015) or dialysis therapy (Karamanidou et al., 2014). How ‘burdensome’ a treatment is informs peoples’ needs and concerns about the treatment (Horne, 2003). ‘Treatment burden’ for patients with long‐term conditions is identified as the loss of freedom and independence for some patients (Demain et al., 2015). Relational disruptions can also occur due to treatment burden, including strained family and social relationships and feeling isolated (Demain et al., 2015). Some of these aspects were reported by the patients in this study, in terms of the time commitment required to attend for transfusions or necessary life‐alterations (e.g., travel). Thus, it is important to continue to explore the construct of ‘Burden’ in more detail with transfusion patients as treatment burden may impact a haematology patient's life to a greater extent than shared by patients in the current interviews.

Strengths of this study include the concurrent investigation of both patients’ and HCPs’ transfusion perceptions from an under‐researched transfusion context, where participants across two NHS sites reported similar perceptions. Patients were interviewed during or just prior to their transfusion, minimising recall bias and potentially heightening the vivid and actual account of their beliefs and experiences. Strengths also include organizing the themes of perceptions into patient and HCP‐specific transfusion conceptual models. This helps to portray relationships between constructs. Limitations, however, include the lower than anticipated inter‐rater reliability scores for the deductive coding and lack of secondary ratings for the triangulation analysis, increasing risk of subjective bias. However, multiple raters and co‐researchers observed and contributed to the final thematic coding and exemplary quotes have been provided as supporting evidence (Nowell et al., 2017).

Future research could extend this study by utilising the applicable patient or HCP conceptual model in a larger quantitative study, translating the themes into questionnaire domains to investigate prevalence of the perceptions on a larger scale and relationships between the themes. This could impact the content of the themes in the existing model as well as formulating themes specific to this health and illness context. The models could also be used as future qualitative frameworks to investigate whether other patient and HCP groups hold similar beliefs about transfusion, such as sickle‐cell patients who often face barriers to attend hospital or disclosing information to HCPs (Maxwell et al., 1999). Principles of open science could be more strongly adhered to in future qualitative studies through gaining consent to publish anonymised participant transcripts or larger extracts from interviews in online journal articles or patient targeted websites (Riley et al., 2019).

Through understanding patients’ perceptions, greater support could be offered to patients through service enhancements. This study indicated that patients may benefit from being more informed about their transfusion, such as the risks of blood products used and being more involved in the decision‐making process. If HCPs are more aware of patients’ perceptions, they may seek to investigate whether patients hold common misperceptions and may, in some cases, challenge these. This may enable patients to cope better and support them with the decision‐making process (e.g., risk vs. benefit evaluations), which may enhance treatment adherence (Horne & Weinman, 1999).

In conclusion, this study has identified a broad range of patients’ and HCPs’ transfusion perceptions revealing challenges for this context such as the burden of receiving repeated transfusion and transfusion‐associated risks. In spite of this, the necessity of transfusion and the benefit of strong links between patients in haematology units and their HCPs were highlighted. HCPs also appeared motivated to improve their transfusion practice, which was reportedly stretched in terms of capacity (e.g., full transfusion appointment slots) with HCPs reporting a high level of goodwill to ensure patients’ well‐being. Service improvements, such re‐designed patient consultations to enhance patient involvement in shared decision‐making or patient education initiatives to are likely to enhance patients’ experiences further.

## CONFLICTS OF INTEREST

The authors declare no conflict of interest.

## AUTHOR CONTRIBUTIONS

**Brittannia Volkmer:** Conceptualization; Data curation; Formal analysis; Investigation; Methodology; Project administration; Resources; Software; Writing – original draft. **Fabiana Lorencatto:** Conceptualization; Methodology; Project administration; Supervision; Validation; Visualization; Writing – review & editing. **Simon J. Stanworth:** Conceptualization; Project administration; Resources; Supervision; Validation; Visualization; Writing – review & editing. **Shashivadan P. Hirani:** Supervision; Validation; Visualization; Writing – review & editing. **Jill J. Francis:** Conceptualization; Funding acquisition; Methodology; Supervision; Validation; Visualization; Writing – review & editing.

## Data Availability

Transcripts are unable to be uploaded to a data sharing repository as informed consent for this purpose was not obtained from participants.

## APPENDIX 1

### Interview topic guides

#### Patients

Thank you for agreeing to take part in the study. As described in the information sheet, I am interested to hear your views about blood transfusion, so please be as open with me as you wish to.

Are you ok with me to start recording the conversation?

*If telephone participant:* As this is a telephone interview, could you please confirm for the recording that you agree to take part in the study?

Ok, thank you… Are you ready to begin?

1. Could you talk to me about your experience of receiving blood transfusions?
  - Prompt: how did you become aware that you needed transfusions?
2. How often do you receive transfusions?
  - *Prompt if regular recipient:* how long have you been receiving transfusions?
  - *Prompt if regular recipient:* how is it decided each time which type of transfusion you will get? (or if you will receive the scheduled transfusion)
  - *Prompt if regular recipient:* What are your thoughts about how often you receive transfusions? (For example: receive too much, not enough)
3. Have you ever been offered any alternatives to transfusion?
  - *Prompt:* what are your thoughts about this?
  - *Prompt if regular recipient:* Has your experience of receiving transfusions changed over time?
4. How much would you say you need transfusions to protect your health?
  - Probe: distinction between protecting health presently or long‐term
5. Are there any downsides for you in receiving transfusions?
  - *Prompt if yes:* Could you tell me more about this?
  - *Further prompt:* How concerned or worried are you about this?
6. What are the benefits of transfusion for you?
  - Probe: distinctions between expected benefits vs. instant and later benefits?
  - *Prompt:* was that what you expected the benefits to be?
7. What do you think are your doctors’ or nurses’ views of transfusion?
  - *Prompt: …* and have you spoken to other patients about their views of having transfusions?
  - *Further prompt:* could you tell me a little bit more about this? (For example: were the discussions helpful for you, did you find that other patients shared your views?)
8. How involved were you in making decisions about your transfusion(s)?
  - *Prompt:* how happy were you about this level of involvement?
9. Could you describe any other ways that you might like to be more involved in your transfusion(s)? (For example: in treatment decision‐making, other treatment discussions, involvement in safety behaviours like wristband or blood bag checking)
  - *Prompt:* what are your reasons for this?
10. Do you have thing else that you would like to tell me about your thoughts on blood transfusion?

This is the end of the interview, thank you for your time. Would you like to be sent a copy of the study results?

If yes: enquire how best to provide the patient with the results, collect details below.

#### Health professionals

Thank you for agreeing to take part in the study about your perceptions of blood transfusion. Some questions may be unfamiliar, for example, rather than looking to understand your content expertise, I am interested to more so understand your beliefs or thoughts, as a healthcare professional, about blood transfusion. What you share with me in the interview is confidential, so please be as open with me as you wish to be.

Are you ok with me to start recording the conversation?

*If telephone participant:* As this is a telephone interview, could you please confirm for the recording that you agree to take part in the study?

Ok, thank you… Are you ready to begin?

1. Can I begin by asking, just briefly, what is your role in the blood transfusion process?
2. Can you tell me about the last time you [prescribed / administered] a patient a blood transfusion? (For example: what happened? were they a regular patient of yours? Did you discuss the transfusion with the patient or other colleagues? … )
3. How much do you think haematology patients need transfusions to protect their health?
  - Probe: distinction between protecting health presently or long‐term
  - *Prompt:* how is this need often determined and communicated to the patient?
4. Can I just ask, what are your thoughts on how often transfusions are prescribed to haematology patients? (For example: prescribed too much, not enough)
5. How much are transfusion alternatives considered for haematology patients and offered to them?
  - (Prompt: are there some reasons that transfusions may be the preferred or only way forward?)
6. Do you think that there are any downsides for haematology patients in having transfusions?
  - *Prompt if yes:* Could you tell me more about this?
  - *Prompt*: Do any of these downsides concern or worry you?
  - *Follow up prompt:* Would you be able to tell me a little bit more about this?
7. What are the benefits of transfusion for haematology patients? Prompt: are there other benefits that you would expect for the patients but don’t really see or hear about?
8. What do you think are your patients’ views of transfusion?
9. To what extent do you think your views about transfusions are shared by your clinical colleagues?
10. Have your views about transfusion changed during the course of your practice?
  - *Prompt if yes:* Could you tell me more about this?
11. How much do you involve patients in their transfusions? (For example: in [treatment decision‐making], involvement in safety behaviours like wristband or blood bag checking)
12. What are your views about involving patients in blood transfusion?
13. Do you have anything else that you would like to tell me?

This is the end of the interview, thank you for your time. Would you like to be sent a copy of the study results?

If yes: enquire how best to provide the HCP with the results, collect details below.

## APPENDIX 2

### Data saturation table for haematology interview study

**Table:**

Subthemes present in the final three patient interviews	N of patients reporting data to these subthemes	Participants 12–14 (X indicates participant contributed to this theme)			Final patients reporting data to subtheme
	(interviews 1–11)	12	13	14	
*Patients*					
Awareness of risk/safety (3 of 5 subthemes)					
Discomfort and illness during or post‐transfusion	5	X		X	7
Health risks from high iron levels	2	X	X		4
Potential infection or reaction risk	4			X	5
Health benefits (5 of 6 subthemes)					
Boosting blood levels	6		X	X	8
Keep going with daily life	5	X	X		7
Relief of symptoms such as tiredness	6	X			7
Anticipated benefits	4		X	X	6
Can take time to feel benefit of transfusion	1		X		2
	**(interviews 1–11)**	**12**	**13**	**14**	
Negative emotions (6 of 8 subthemes)					
No concerns or worries with transfusions	8		X	X	10
Attempts to manage worries and fear	6	X	X	X	9
Gratitude that transfusions possible	3	X		X	5
Relaxed during transfusion appointments	4			X	5
Receiving transfusions unpleasant	3	X			4
Positive emotions of not needing transfusion	1	X			2
Alternatives (1 of 4 subthemes)					
Alternatives considered or already in use	5	X		X	7
Necessity (3 of 5 subthemes)					
Transfusions required as a current and long‐term supportive treatment	7	X	X	X	10
Need established by HCPs and clinical indicators	7	X			8
Need for transfusion apparent through symptoms	6	X	X		8
	**(interviews 1–11)**	**12**	**13**	**14**	
Involvement in decision making (5 of 7 subthemes)					
Willing acceptance of transfusions	6	X			7
Confronted with limited or no choice	5		X		6
Transfusion offered with patient involvement in choice	4	X	X	X	7
Routine ‘automatic’ treatment	2			X	3
More frequent transfusions would be resisted	1	X			2
Burden (4 of 4 subthemes)					
Transfusion part of routine life	5		X	X	7
Transfusions are inconvenient	4	X			5
Attendance not a great burden	3		X		4
Life restrictions, travel	2	X	X		4
Social connection (3 of 5 subthemes)					
Patient involvement generally positive	4	X		X	6
	**(interviews 1–11)**	**12**	**13**	**14**	
Lack of interaction or activity during transfusions	4			X	5
Interaction with other patients	4	X	X		6
HCPS					
Awareness of risk/safety (6 of 6 subthemes)					
Risks mitigated by safe transfusion practices	9	X	X	X	12
Risks and benefits established with patients	7	X		X	9
Iron overload considered a key risk	5	X	X		7
Infections, antibodies and reactions risks	6			X	7
Short and long‐term medical and psychological impact	4	X	X		6
Risk of not providing a transfusion	2	X	X	X	5
Alternatives (4 of 4 subthemes)					
Alternatives considered or already in use	8		X	X	10
	**(interviews 1–11)**	**12**	**13**	**14**	
No alternatives, transfusion the only option	5		X	X	7
Support for greater consideration and use of alternatives	4		X	X	6
Committed to giving regular transfusions once started	2		X		3
Burden (2 of 2 subthemes)					
Anticipated attendance burden for patients	9	X		X	11
Transfusion has become a part of patient's routine life	5		X	X	7
Health benefits (6 of 6 subthemes)					
Symptom improvement, making patients feel better	9	X	X		11
Supportive care to carry on with normal daily living	9		X		10
Benefit lasts a limited time only	7		X		8
Shared HCP agreement of transfusion benefits	4			X	5
	**(interviews 1–11)**	**12**	**13**	**14**	
Patient questioned on benefits to provide / continue transfusions	4		X		5
Some risk‐benefit for patients questionable	2		X		3
Necessity (4 of 5 subthemes)					
Transfusions support chemotherapy or used to treat anaemia	9	X	X	X	12
Transfusions are vital, aiding survival	8		X	X	10
Transfusions given to protect health	7		X		8
Necessity established using clinical and patient factors	7			X	8
Negative emotions (5 of 7 subthemes)					
Practice concerns and frustrations	9			X	10
Patient anxiety and upset with receiving regular transfusions	8	X	X		10
	**(interviews 1–11)**	**12**	**13**	**14**	
Concern about downsides of transfusions for patients	8	X	X		10
Patients’ unexpressed potential negative emotions	7	X	X		9
Upset in witnessing patients’ worsening health or death	2			X	3
Involvement in decision making (6 of 8 subthemes)					
HCPs advocate and involve patients in decisions	10	X	X	X	13
Team decision on transfusion prescription	12			X	13
Patient autonomy in their own transfusion decisions	9	X	X		11
Individual transfusion regime for each patient	8		X	X	10
Transfusions prescribed appropriately using guidelines	5	X	X	X	8
Barriers to discussing transfusion or obtaining consent	3		X	X	5
	**(interviews 1–11)**	**12**	**13**	**14**	
Organisational factors (5 of 5 subthemes)					
Solutions needed to improve processes and ease capacity strain	6		X		7
Solutions needed to enhance communication	5		X		6
High and costly blood use for hospital	5	X			6
Constraints to greater discussion of patients’ views	5		X		6
Complicated management of transfusion slots	4			X	5
Stability and variability of transfusion perceptions (3 of 3 subthemes)					
Views consistent and similar to colleagues	6			X	7
Views broadened through haematology exposure	4	X	X	X	7
Patients’ transfusion perceptions variable	1			X	2

## APPENDIX 3

### Themes of patients’ perceptions with frequencies of patients reporting to each theme and example quotations

**Table:**

Themes	Frequency (n patients)	Example quotations
Awareness of risk/safety		
Discomfort and illness during or post‐transfusion	7	‘there's always situations that I was here hours waiting for blood and I ended up receiving it, but the following day I was in bed all day’ (Patient 12, Aplastic Anaemia, Site 2)
Potential infection or reaction risk	5	‘basically, you kind of just think, you know, ‘is this gonna be the transfusion that might cause me to have a reaction?’ or, ‘is this the unit that's going to cause something in the future?’ but you can't really live like that, so you don't really think about it too much really, it doesn't stop me having them’ (Patient 8, other Anaemia, Site 1)
No experienced negative consequences	5	‘I feel quite good really, I’m not having any side‐effects or anything like that which is very good’ (Patient 6, Myelofibrosis female, Site 1)
Health risks from high iron levels	4	‘because the more blood I receive, the more the iron level in my blood goes up’ (Patient 12, Aplastic Anaemia, Site 2)
Caution needed, blood should be used appropriately	2	‘there's many, many, many issues of blood, you know, if the haemoglobin's dropped or loss of blood, or that sort of thing, but er, I think people need to be a bit more careful’ (Patient 8, other Anaemia, Site 1)
Health benefits		
Boosting blood levels	8	‘well normally it/ well, it depends, erm, how low the haemoglobin is, the lower it is then the benefit is quicker because you're being topped up but’ (Patient 14, Inherited Anaemia, inc Thalassemia, Site 2)
Keep going with daily life	7	‘well I wouldn't be able to get about, I'd be fighting for breath and all that if I didn't have it, you know, I wouldn't be able to do anything I do now. I can walk about’ (Patient 7, Myelodysplasia, Site 1)
Relief of symptoms such as tiredness	7	‘when I received blood transfusion after that, I rest a bit and then I feel strong but before I feel weak’ (Patient 10, other Anaemia, Site 2)
Anticipated benefits	6	‘I just feel as though that if I got a transfusion with fresh blood, surely I’ve got to feel better from it, I think so’ (Patient 1, Myeloma, Site 1)
Patient convinced of benefit by significant others	3	‘everyone was telling me I looked better and that in itself made me feel better as well I suppose’ (Patient 1, Myeloma, Site 1)
Can take time to feel benefit of transfusion	2	‘this is a benefit, the blood, but sometimes it takes a while to kick in, it's like if you're taking medication from the doctor, sometimes it takes, erm, a while to kick in’ (Patient 11, Lymphoma (CLL) and a second haematological condition, Site 2)
Necessity		
Transfusion prolonging life and aiding survival	11	‘oh yes, it just seems to me as being essential, erm, as much as say needing oxygen in the air is an essential, without the transfusions I wouldn't be here’ (Patient 4, Aplastic Anaemia, Site 1)
Transfusions required as a current and long‐term supportive treatment	10	‘they gave me two courses of ATG, it's an immunogloubulin … the second time, I didn't have any response. Since then, since, … I've been receiving transfusions’ (Patient 12, Aplastic Anaemia, Site 2)
Need established by HCPs and clinical indicators	8	‘if it is lower than 80, they said I need a blood transfusion … sometimes it depends, they keep seeing the blood test, decided if I need to have a blood transfusion’ (Patient 10, other Anaemia, Site 2)
Need apparent through symptoms	8	‘yeah, when it drops, I feel a bit tired, but/ you feel it, dizzy, a little bit dizzy, tiredness, shortness of breath, sometimes/ I try to manage it, but/’ (Patient 9, Acquired Haemolytic Anaemia, Site 2)
Speculation and doubt over pending transfusion prescription	7	‘Erm, last week I had two units of blood ∙ but this week ∙ it's only like the platelets, so possibly next week it'll just be platelets and one unit of blood’ (Patient 2, Aplastic Anaemia, Site 1)
Negative emotions		
No concerns or worries with transfusion	10	‘no [concerns or worries], cause there's no point worrying about something I can't really do anything about’ (Patient 8, other Anaemia, Site 1)
Attempts to manage worries and fears	9	‘so, it doesn't look, it looks ok for you for a minute, and then you start to think about something else but that one you have to be on the positive side all the time, maybe not keep saying ‘that's not good'‘ (Patient 9, Acquired Haemolytic Anaemia, Site 2)
Relaxed during transfusion appointments	5	‘I come regularly, erm, to be honest it's a nice, this sounds really weird but, you know, it's a day where, I, I fully relax because I'm just having my blood and I'm not thinking of anything else’ (Patient 14, Inherited Anaemia, inc Thalassemia, Site 2)
Gratitude that transfusions possible	5	‘I'm very grateful that there is blood available for me and other patients, erm, and I appreciate that hugely’ (Patient 14, Inherited Anaemia, inc Thalassemia, Site 2)
Receiving transfusions unpleasant	4	‘everybody hates having them, they all hate the cannula's in’ (Patient 2, Aplastic Anaemia, Site 1)
Concern of transfusion dependency	4	‘sometimes, erm, I feel scared … of course if you see blood, you think, you think to yourself that it's bad’ (Patient 10, other Anaemia, Site 2)
Positive emotions of not needing transfusion	2	‘there was a wonderful telephone message and it said [Mr/Mrs surname], we don't want to see you today and so everyone cheered you know. I, I didn't come in, which was brilliant’ (Patient 4, Aplastic Anaemia, Site 1)
Perception that doctors dislike prescribing transfusions	1	‘no, most of the doctors like, they, they always hate giving transfusions to someone …’ (Patient 9, Acquired Haemolytic Anaemia, Site 2)
Alternatives		
Alternatives considered or already in use	7	‘I was offered the main treatment for this condition, is the bone marrow transplant, but I, I'm not really keen to do that, cause it's, I've, erm, you know, the side effects of treatment …’ (Patient 12, Aplastic Anaemia, Site 2)
No alternatives, transfusion only option	5	‘yeah, I would just fade away, yeah, cause there's nothing else that can help me’ (Patient 8, other Anaemia, Site 1)
Patient's body can correct depleted cells	3	‘hopefully, erm, with erm, not needing platelets, might just be my body saying ‘I’ll have a go now’’ (Patient 4, Aplastic Anaemia, Site 1)
Patient preference for alternatives	3	‘I wish they'd/ sometimes I wish that I didn't have to have it done. I wish they could just give you like a tablet and something like that’ (Patient 11, Lymphoma (CLL) and a second haematological condition, Site 2)
Involvement in decision making		
Transfusion offered with patient involvement in choice	7	‘yeah I had choice, I had a choice, yes I will go along with it, or no I won't bother. She gave me that choice as well, ∙ but I was led by her professional advice’ (Patient 1, Myeloma, Site 1)
Deferral of decision making to HCPs	7	‘I just take it for granted that what they're asking me to do, or what they're doing is the correct thing to do’ (Patient 5, Myelodysplasia, Site 1)
Willing acceptance of transfusions	7	‘I suppose I sort of took it in my stride really, think well if they're gonna help me, then you've gotta go along with it, haven't you really, that's how I feel’ (Patient 6, Myelofibrosis female, Site 1)
Confronted with limited or no choice	6	‘They're the experts and they say so, and, like they said ‘if you don't want it, you just die’, ‘you choose to die’, they said, ‘don't bother coming in'‘ (Patient 7, Myelodysplasia, Site 1)
Decision making discussion positive	5	‘[the consultant] does try to explain in simple terms, er, what [he/she] thinks, and the, the whole thing has been optimistic and upbeat … there's never been any pessimism at all, which has been terribly encouraging’ (Patient 4, Aplastic Anaemia, Site 1)
Routine ‘automatic’ treatment	3	‘with it being, you know, a chronic condition, so very long‐term, so it's almost automatic regular treatment’ (Patient 14, Inherited Anaemia, inc Thalassemia, Site 2)
More frequent transfusions would be resisted	2	‘I try to avoid as well because the more blood I receive, the more the iron level in my blood goes up’ (Patient 12, Aplastic Anaemia, Site 2)
Social connection		
Patient involvement generally positive	6	‘I think I’m quite involved in everything that does go on, erm, they do keep you ∙ up to date and everything’ (Patient 2, Aplastic Anaemia, Site 1)
Interaction with other patients	6	‘we know a lot of people here so we have chats, things like that – so it's like a little family really’ (Patient 6, Myelofibrosis, Site 1)
Lack of interaction or activity during transfusions	5	‘Well except in my case because being partially sighted, I can't read, can't see peoples’ faces, so er, I'm just sat here for about eight to ten hours, just looking at the wall more or less’ (Patient 5, Myelodysplasia, Site 1)
Curiosity and appreciation for blood donors	3	‘you know ‘what do you think about the blood?’ and erm, I think, I think about the blood, that it came from somebody, just curious to know a little bit more about the person’ (Patient 4, Aplastic Anaemia, Site 1)
Support from family or primary care HCPs	2	‘when we approach the doctor, my family member: ‘ask this one, ask this one’, he/she is like/ so, which is a big help, but like me, I don't know what to ask’ (Patient 10, other Anaemia, Site 2)
Burden		
Transfusion part of routine life	7	‘I carry on completely normal, normal life with the, you know, the odd transfusion every now and again, yeah’ (Patient 8, other Anaemia, Site 1)
Transfusions are inconvenient	5	‘I think it's too often because being in hospital twice a week, minimum twice a week/ cause the week I receive blood, I have to come three times, and it's exhausting’ (Patient 12, Aplastic Anaemia, Site 2)
Life restrictions, travel	4	‘it stops you from, if you ever wanted to go to another country, make a life in another country, it's a downside of it, the whole thing, be very difficult to do that’ (Patient 13, Inherited Anaemia, inc Thalassemia, Site 2)
Attendance not a great burden	4	‘it's not a burden to me, I don't live too far away’ (Patient 4, Aplastic Anaemia, Site 1)
Distinguishing between blood products		
Knowledge gaps for platelets	2	‘I don't really understand what platelets do, I know, I know that they, that they repair the damage in the body, and works like that erm, ∙∙ well I am not a doctor am I’ (Patient 5, Myelodysplasia, Site 1)
Positive perception of platelets	2	‘it's just that one's shorter than the other. The erm, platelets only take about 20 min to half an hour’ (Patient 2, Aplastic Anaemia, Site 1)
Distinction between non irradiated and irradiated blood	1	‘all blood transfusions are different, but this is irradiated blood transfusion’ (Patient 11, Lymphoma (CLL) and a second haematological condition, Site 2)

Themes of HCPs’ perceptions with frequencies of HCPs reporting to each theme and example quotations.

**Table:**

Themes	Frequency (n HCPs)	Example quotations
Awareness of risk / safety		
Risks mitigated by safe transfusion practices	12	‘…it's all quite safe, erm, in terms of, em, getting the right blood product for the patient, cause it's different steps in safeguarding steps to do that’ (HCP 10, Senior House Office, Site 2)
Risks and benefits established with patients	9	‘so you just try to reassure it's a rarity, for you to obviously get anything from transfusion, and erm, reactions is the one that commonly comes up as well, they worry about reacting to, cause, ‘does it have any side effects?’ is the common question I get asked’ (HCP 8, Nurse, Site 2)
Iron overload considered a key risk	7	‘the downside is if they continue to have lots and lots and lots of blood transfusions they will become / have high iron levels … so then you have the problem of the liver being affected because high ferritin, so that's the downside of it’ (HCP 2, Nurse, Site 1)
Infections, antibodies and reactions risks	7	‘um, long‐term wise I think it is probably not good for them because the more you get transfusion, the more becomes/ they develop the risk of having antibodies, er, and that can be very bad for them in the long run because you are/ every time we cross‐match their blood products, it becomes rarer and rarer’ (HCP 9, Senior Charge Nurse, Site 2)
Short and long term medical and psychological impact	6	‘they're also concerned about getting addicted to blood transfusions’ (HCP 12, Clinical Psychologist, Site 2)
Risk of not providing a transfusion	5	‘they've got Leukaemia or MDS (Myelodysplasia Syndromes) and they just, and they know that this is all you can do, they just need to have their transfusion every how ever many weeks, and if you're not providing a chair for them, then they could end up on our medical assessment unit’ (HCP 2, Nurse, Site 1)
Health benefits		
Symptom improvement, making patients feel better	11	‘Yeah, so largely it erm, taking away the tiredness and lethargy, which is, er, a symptom of patients that are anaemic, er, and for some of them improving their symptoms of shortness of breath on exertion’ (HCP 7, Specialist Registrar, Site 1)
Supportive care to carry on with normal daily living	10	‘it's about trying to give them a quality of life, so trying to work out what's the best, you know, what's the best, amount of blood to give them if you like’ (HCP 2, Nurse, Site 1)
Benefits last a limited time only	8	‘… it's really tricky to say to that person, or to their relatives ‘right, we don't think you're benefitting from blood anymore’ because they know if they don't have the blood they will die’ (HCP 3, Specialty doctor (Haematology), Site 1)
Shared HCP agreement of transfusion benefits	5	‘yeah I think generally we all share the same view that actually it's benefiting the patient’ (HCP 9, Senior Charge Nurse, Site 2)
Patient questioned on benefits to provide / continue transfusions	5	‘…when we see them in clinic or when we see them in the day unit, um, and if we find they're having symptomatic benefit from it, you know then we carry on with it’ (HCP 3, Specialty doctor, Site 1)
Some risk‐benefit for patients questionable	3	‘I think quite, some of my patients who've maybe only got a slight anaemia, sort of eight to nine, haven't felt any better on transfusions, I think they'd rather have the slightly low count, so I think it really depends how low their haemoglobin goes’ (HCP 13, Consultant Haematologist, Site 2)
Necessity		
Transfusions support chemotherapy or used to treat anaemia	12	‘as I've said, it's the only way, with a lot of these people, it's the only thing that's keeping them alive, or it's the thing that's allowing them to have treatment, that's hopefully going to keep them alive’ (HCP 8, Nurse, Site 2)
Transfusions are vital, aiding survival	10	‘it is palliative technically, it is because we are keeping them alive cause if we didn't transfuse them, they will die’ (HCP 5, Consultant Haematologist, Site 1)
Transfusions given to protect health	8	‘if somebody has got a really low haemoglobin, they need the blood, cause obviously they're struggling, sometimes they're breathless, it puts a strain on the heart’ (HCP 4, Transfusion Practitioner, Site 1)
Necessity established using clinical and patient factors	8	‘it (frequency of the transfusions) depends on their haemoglobin, the clinical symptoms, and the data, I mean, the blood results’ (HCP 11, Haematology Specialist Nurse, Site 2)
Patients rely on and express need for transfusion	5	‘but other patients are so fixated by it, and it is keeping them going, that I think sometime it's, I think, they really rely on it’ (HCP 1, Haematology Specialist Nurse, Site 1)
Negative emotions		
Patients’ unexpressed potential negative emotions	12	‘Erm, I'm not quite sure though, how they exactly feel about their specific transfusion going in at the time, whether they're anxious, or? whether they feel happy, or? I dunno’ (HCP 10, Senior House Office, Site 2)
Patient anxiety and upset with receiving regular transfusions	10	‘they do get really worried if they come in and their haemoglobin's low, they immediately start thinking something bad's happening or worrying that they're going to have to start coming in every week’ (HCP 8, Nurse, Site 2)
Concern about downsides of transfusions for patients	10	‘it is worrying, I mean people, if they've got cardiac problems, they can get chest pain, just really really unwell’ (HCP 2, Nurse, Site 1)
Practice concerns and frustrations	9	‘I do worry here that we don't have enough capacity to get patients who are becoming acutely unwell and there isn't a space for them, and that really frightens me’ (HCP 4, Transfusion Practitioner, Site 1)
HCPs’ strategies to reduce patient anxiety	6	‘[transfusion] is not treating the underlying cause and therefore their transfusion requirement will go up and at some point, we will stop. Because we warn them early that that's gonna happen; when that does happen they're less shocked’ (HCP 7, Specialist Registrar, Site 1)
Patients perceiving transfusion positively, as a lifeline	5	‘I think people tend to be positive about it cause I think they get the fact that, you know, this is, this is all we've got for you, but actually it does work, you know’ (HCP 3, Specialty doctor, Site 1)
Upset in witnessing patients’ worsening health or death	3	‘yeah, it's quite distressing, it's quite distressing, especially when you've known somebody for a number of years and then it gets to that point, you do sort of kind of think ‘oh why are they coming in’’ (HCP 5, Consultant Haematologist, Site 1)
Alternatives		
Alternatives considered or already in use	10	‘most of these patients that are on such regular transfusion programmes are on EPO (erythropoietin) are on iron, are on all the other kind of alternatives to blood that they can be on and despite that are still requiring a blood transfusion’ (HCP 10, Senior House Office, Site 2)
No alternatives, transfusion only option	7	‘I think as clinicians, people always weight that up and I think it's very difficult because I think there genuinely isn't an alternative … I just think there really isn't an alternative unfortunately’ (HCP 4, Transfusion Practitioner, Site 1)
Support for greater consideration and use of alternatives	6	‘in terms of the patients who require regular top up transfusions, it doesn't seem like there's much research done into alternative therapies and things and so we just readily assume ‘oh we'll do bloods’ as that's all we know, that's all we're readily exposed to’ (HCP 9, Senior Charge Nurse, Site 2)
Committed to giving regular transfusions once started	3	‘So once you get someone in to having transfusion, you can't, if you could/ you switch the on switch, but you can't flick it off again and it usually ends up with that person being admitted to the main hospital with some infection…’ (HCP 2, Nurse, Site 1)
Involvement in decision making		
HCPs advocate and involve patients in decisions	13	‘… other patients want to take control of it and not be told what to do, so you have to be, erm, I think you have to be flexible about that’ (HCP 7, Specialist Registrar, Site 1)
Team decision on transfusion prescription	13	‘the nursing staff had reviewed the bloods and felt that they needed a blood transfusion, but then also/ ultimately the decision is with, um, with the doctor’ (HCP 1, Haematology Specialist Nurse, Site 1)
Patient autonomy in their own transfusion decisions	11	‘I think he/she pushes it quite far, so he/she might avoid coming out for a transfusion to see if his/her haemoglobin gets better’ (HCP 3, Specialty doctor, Site 1)
Individual transfusion regime for each patient	11	‘we try and individualise transfusion practice per patient, so if you have a patient whose haemoglobin drops down to below, I don't know, 70, on every two weeks, that's how often you give them their blood, so it is individualised’ (HCP 5, Consultant Haematologist, Site 1)
Transfusions prescribed appropriately using guidelines	12	‘to actually have something like that to say ‘well I am following NICE guidance, therefore, I've got this huge! weight of evidence behind me, so I feel confident to make that decision'‘ (HCP 4, Transfusion Practitioner, Site 1)
Deferral of decision making to HCPs	6	‘I think a lot of patients rightly so place their trust in the medical team, the nurses and the doctors, so if somebody said they need a transfusion, I think it's very few and far between patients that say no’ (HCP 14, Specialist Registrar, Site 2)
Barriers to discussing transfusion or obtaining consent	5	‘it is not always possible (obtaining and documenting verbal consent), especially in patients who are either unable to give consent or, you know, patients who are not in a state, because of the physical condition to give consent’ (HCP 3, Specialty doctor, Site 1)
Tendency towards providing transfusion	4	‘if they're very symptomatic with it, and even if the level is slightly off the baseline but they're very symptomatic then we like to give it’ (HCP 10, Senior House Office, Site 2)
Burden		
Anticipated attendance burden for patients	11	‘if they're really regular I suppose it's the fact they're having blood test and they're here, and they're seeing the doctors, and you do, it must be quite hard, patients must feel they're like here all the time almost’ (HCP 1, Haematology Specialist Nurse, Site 1)
Transfusion's become a part of patient's routine life	7	‘I think for some, it's just like a way of life for them, em, it's just something that they do and they know other patients who are on a similar sort of path and then they're quite pally with them…’ (HCP 9, Senior Charge Nurse, Site 2)
Organisational factors		
Solutions needed to improve processes and ease capacity strain	7	‘… we're trying to organise transfusions, at short notice, for our patients at short notice on the day unit and ˆ they've got no capacity to deliver the transfusion’ (HCP 6, Consultant Haematologist, Site 1)
Constraints to greater discussion of patients’ views	6	‘there's only one of you, and seven patients, it's not always easy to sit with the patient, discuss any concerns or issues or just have a general conversation’ (HCP 8, Nurse, Site 2)
Solutions needed to enhance communication	6	‘I think there's the other side about educating staff as well about blood transfusion, you know, and how to define risks and you know, encouraging them to discuss with the patients’ (HCP 2, Nurse, Site 1)
High and costly blood use for hospital	6	‘I mean blood transfusion can work, but, um, it is quite a bit more costly both for the hospital and for the patient's time’ (HCP 12, Clinical Psychologist, Site 2)
Complicated management of transfusion slots	5	‘so they send me a letter or an e‐mail or something saying ‘this person is going to need blood product support and then we're like ‘well, where are we gonna put them in then?’ (HCP 3, Specialty Doctor, Site 1)
Stability and variability of transfusion perceptions		
Views consistent and similar to colleagues	7	‘I think there's a general agreement about the use of transfusions for this patient group… yeah, I'd say we pretty much all have the same view’ (HCP 10, Senior House Office, Site 2)
Views broadened through haematology exposure	7	‘before blood transfusions were a one‐off sort of thing … since being here, it's actual haematology conditions that require regular blood transfusions, so I see a different side now, and I see, erm how reliant people are on them’ (HCP 5, Consultant Haematologist, Site 1)
Patients’ transfusion perceptions variable	2	‘I think it's very variable, I don't think there's like one general consensus across the patients definitely’ (HCP 14, Specialist Registrar, Site 2)
Supportive relationships		
HCPs approachable and bond with patients	5	‘… one of our regulars died in, in the week on the ward, and it's upsetting because you know, we get to know them so well and all about them, they talk to you a lot and erm, and it gets quite personal’ (HCP 3, Specialty Doctor, Site 1)
Efforts to increase patient comfort in unit	4	‘they are often coming in with someone, erm, that you know, they bring someone with them … just to ensure that they've got relatives, or they've got people involved’ (HCP 8, Nurse, Site 2)

## APPENDIX 4

### Patient and HCP themes with agreement ratings

**Table:**

Theme header	Patient (P) or HCP	Sub‐theme label	Agreement rating
Burden	P	Transfusions are inconvenient	Agreement with ‘*Anticipated attendance burden for patients’*
	P	Attendance not a great burden	Disagreement
	HCP	Anticipated attendance burden for patients	
Burden	P	Transfusion part of routine life	Agreement with ‘*Transfusion has become a part of patient's routine life’*
	P	Life restrictions, travel	Disagreement
	HCP	Transfusion has become a part of patient's routine life	
Distinguishing blood products	P	Knowledge gaps for platelets	Silent
Distinguishing blood products	P	Positive perception of platelets	Silent
Distinguishing blood products	P	Distinction of irradiated blood	Silent
Social connection+Decision‐ making	P	Patient involvement generally positive	Partial agreement with ‘*HCP advocates and involves patients in decisions’*
	P	Transfusion offered with patient involvement in choice	Partial agreement
	HCP	HCP advocates and involves patients in decisions	
Social connection	P	Interaction with other patients	Silent
Social Connection	P	Lack of interaction or activity during transfusions	Silent
Social Connection	P	Curiosity and appreciation for blood donors	Silent
Social Connection	P	Support from family or primary care HCPs	Silent
Supportive relationships	HCP	HCPs approachable and bond with patients	Silent
Supportive relationships	HCP	Efforts to increase patient comfort in unit	Silent
Organisational Constraints	HCP	Solutions needed to improve processes and ease capacity strain	Silent
Organisational Constraints	HCP	Constraints to greater discussion of patients’ views	Silent
Organisational Constraints	HCP	Solutions needed to enhance patient‐HCP and team communication	Silent
Organisational Constraints	HCP	High and costly blood use for hospital	Silent
Organisational Constraints	HCP	Complicated management of transfusion slots	Silent
Stability and variability of transfusion perceptions	HCP	Views on transfusion broadened through haematology exposure	Silent
Stability and variability of perceptions	HCP	Views consistent and similar to colleagues	Silent
Stability and variability of perceptions	HCP	Patients’ transfusion perceptions variable	Silent
Awareness of risk/Safety	P	Discomfort and illness during or post‐transfusion	Agreement with ‘*Short and long term medical and psychological impact*’
	P	No experienced negative consequences	Disagreement
	HCP	Short and long term medical and psychological impact	
Awareness of risk/Safety	P	Health risks from high iron levels	Agreement
	HCP	Iron overload considered a key risk	
Awareness of risk/Safety	P	Potential infection or reaction risk	Agreement
	HCP	Infections, antibodies and reactions risks	
Awareness of risk/Safety +Involvement in decision‐making	P	Caution needed, blood should be used appropriately	Agreement
	HCP	Transfusions prescribed appropriately using guidelines	
Awareness of risk/Safety	HCP	Risks mitigated by safe transfusion practices	Silent
Awareness of risk/Safety	HCP	Risks and benefits established with patients	Silent
Awareness of risk/Safety	HCP	Risk of not providing a transfusion	Silent
Necessity	P	Transfusions used as a current and long‐term supportive treatment	Partial agreement
	HCP	Transfusions support chemotherapy or used to treat anaemia	
Necessity	P	Transfusion prolonging life and aiding survival	Agreement
	HCP	Transfusions are vital, aiding survival	
	HCP	Transfusions given to protect health	Agreement with ‘*Transfusion prolonging life and aiding survival*’
Necessity	P	Need for transfusion apparent through symptoms	Partial agreement
	HCP	Patient reliance and expressed need for transfusion	
Necessity	P	Speculation and doubt over pending transfusion prescription	Silent
Necessity +Involvement in decision‐making	P	Need established by HCPs and clinical indicators	Agreement
	HCP	Team decision on transfusions prescription	
	HCP	Necessity of transfusion established in balance with clinical and patient factors	Agreement with ‘*Need established by HCPs and clinical indicators*’
Health benefits	P	Keep going with daily life	Agreement
	HCP	Supportive care to carry on with normal daily living	
Health benefits	P	Boosting blood levels	Partial agreement with ‘*Symptom improvement*, *making patients feel better’*
	P	Relief of symptoms such as tiredness	Agreement
	HCP	Symptom improvement, making patients feel better	
Health benefits	P	Can take time to feel benefit of transfusion	Agreement
	HCP	Benefits last a limited time only	
Health benefits	P	Anticipated benefits	Silent
Health benefits	P	Patient convinced of benefit by significant others	Silent
Health benefits	HCP	Shared HCP agreement of transfusion benefits	Silent
Health benefits	HCP	Some risk‐benefit for patients questionable	Silent
Negative emotions	P	No concerns or worries with transfusions	Agreement with ‘*Patients perceiving transfusion positively*, *as a lifeline*’
	P	Gratitude that transfusions possible	Agreement
	HCP	Patients perceiving transfusion positively, as a lifeline	
Negative emotions	P	Receiving transfusions unpleasant	Agreement with ‘*Patient anxiety and upset with receiving regular transfusions’*
	P	Relaxed during transfusion appointments	Disagreement with ‘*Patient anxiety and upset with receiving regular transfusions*’
	P	Concern of transfusion dependency	Agreement
	HCP	Patient anxiety and upset with receiving regular transfusions	
Negative emotions	P	Positive emotions of not needing transfusion	Silent
Negative emotions	P	Attempts to manage worries and fear	Silent
Negative emotions	P	Perception that doctors dislike prescribing transfusions	Partial agreement
	HCP	Concern about downsides of transfusions for patients	
Negative emotions	HCP	Patients’ unexpressed potential negative emotions	Silent
Negative emotions	HCP	Practice concerns and frustrations	Silent
Negative emotions	HCP	HCPs’ strategies to reduce patient anxiety	Silent
Negative emotions	HCP	Upset in witnessing patients’ worsening health or death	Silent
Alternatives	P	Alternatives considered or already in use	Agreement
	HCP	Alternatives considered or already in use	
Alternatives +Involvement in decision‐making	P	No alternatives, transfusion the only option	Agreement with ‘*No alternatives*, *transfusion the only option*’
	P	Confronted with limited or no choice	Agreement
	HCP	No alternatives, transfusion the only option	
Alternatives	P	Patient preference for alternatives	Agreement
	HCP	Support for greater consideration and use of alternatives	
Alternatives	P	Patient's body can correct depleted cells	Silent
Alternatives	HCP	Committed to giving regular transfusions once started	Silent
Involvement in decision‐making +Necessity + Health benefits	P	Willing acceptance of transfusions	Agreement
	HCP	Patient autonomy in their own transfusion decisions	
	HCP	Patient reliance and expressed need for transfusion	Agreement with ‘*Willing acceptance of transfusions’*
	HCP	Patient questioned on health benefits to provide/continue transfusions*	Partial agreement with ‘*Willing acceptance of transfusions’*
Involvement in decision‐making	P	Deferral of decision‐making to HCPs	Agreement
	HCP	Deferral of decision‐making to health professionals	
Involvement in decision‐making	P	Decision‐making discussion positive	Silent
Involvement in decision‐making	P	Routine ‘automatic’ treatment	Silent
Involvement in decision‐making	P	More frequent transfusions would be resisted	Silent
Involvement in decision‐making	HCP	Individual transfusion regime for each patient	Silent
Involvement in decision‐making	HCP	Barriers to discussing transfusion or obtaining consent	Silent
Involvement in decision‐making	HCP	Tendency towards providing transfusion	Silent
